# Ultrasound Imaging of Thoracolumbar Fascia Thickness: Chronic Non-Specific Lower Back Pain versus Healthy Subjects; A Sign of a “Frozen Back”?

**DOI:** 10.3390/diagnostics13081436

**Published:** 2023-04-16

**Authors:** Carmelo Pirri, Nina Pirri, Diego Guidolin, Veronica Macchi, Andrea Porzionato, Raffaele De Caro, Carla Stecco

**Affiliations:** 1 Department of Neurosciences, Institute of Human Anatomy, University of Padua, 35121 Padua, Italy; diego.guidolin@unipd.it (D.G.); veronica.macchi@unipd.it (V.M.); andrea.porzionato@unipd.it (A.P.); rdecaro@unipd.it (R.D.C.); carla.stecco@unipd.it (C.S.); 2 Department of Medicine—DIMED, School of Radiology, Radiology Institute, University of Padua, 35121 Padova, Italy; nina_92_@hotmail.it

**Keywords:** fascia, thoracolumbar fascia, ultrasound examination, thickness, low back pain

## Abstract

The thoracolumbar fascia (TLF) plays an important role in lower back pain (LBP). Recent studies have revealed an association between increases in TLF thickness and reduced TLF gliding in patients with LBP. The purpose of this study was to measure and compare by ultrasound (US) imaging the thickness of the TLF at the bilateral L3 level of the lumbar spine in the longitudinal and transverse axes in chronic non-specific LBP and in healthy subjects. A cross-sectional study was performed using US imaging to measure the longitudinal and transverse axes with a new protocol in a sample of 92 subjects: 46 chronic non-specific LBP patients and 46 healthy participants. The findings for TLF thickness revealed statistically significant differences (*p* < 0.05) in the longitudinal and transverse axes between the two groups. Moreover, in the healthy group, a statistically significant difference was found between the longitudinal and transverse axes (*p* = 0.001 for left and *p* = 0.02 for right), which was not evident in the LBP patients. These findings suggest that the LBP patients lost anisotropy of the TLF, with it becoming homogeneously thicker and losing adaptability in the transversal direction. The US imaging evaluation suggests that TLF thickness behavior points out altered fascial remodelling compared to healthy subjects, a sort of “frozen back”.

## 1. Introduction

Lower back pain (LBP) has received much attention in recent years due to substantial personal and social burden, high disability, and high healthcare expenditure [1,2]. It is one the most common health problems and has a prevalence of 60–85% in the adult population [3,4,5], and the trend has been increasing over the last 20 twenty years [6]. It is known as pain or stiffness in the area between the 12th costal margin and the inferior gluteal folds, sometimes accompanied by the presence of pain in the lower extremities [6]. LBP can be classified as non-specific or mechanical LBP [5]. The first one is defined as back pain of unknown disease; the second one is caused by the pathology of spine, intervertebral discs, and some surrounding soft tissues. Moreover, LBP can be categorized based on the duration of pain. Acute LBP is defined when the pain lasts less than 6 weeks, subacute from 6 weeks up to 3 months, and chronic when it persists for longer than 3 months or is recurring LBP [7].

Different etiologies have been proposed for LBP. Numerous papers have established that LBP is attributed to dysfunction of neuro-musculoskeletal system tissues (e.g., nerve roots, muscles, intervertebral discs, and spinal joints), which can determine and contribute to LBP either individually or collectively [8,9,10,11]. Furthermore, much research in recent years has focused on the role of connective tissue, in particular, the thoracolumbar fascia (TLF) [12,13,14,15].

The TLF is one of the main contributors to the rise of non-specific LBP due to it having a lot of free and capsulate nerve endings, including the Pacini’s and Ruffini’s corpuscles that are present within it [16,17,18]. These structures are tightly connected and embedded in the surrounding fibers of collagen, elastin, and the extracellular matrix (ECM) which fascia is made of. The TLF contains layers of loose and dense connective tissue that independently move across each other to facilitate spine movement [19].

The scientific literature suggests that the pathophysiological mechanism is that TLF inflammation and movement disorders caused by LBP lead to increased stiffness and decreased flexibility of the TLF and consequently limitation of movement in chronic LBP [13,20]. Along these lines, LBP may be a consequence of fibrosis or densification of the fascial layers [13,20].

Ultrasound (US) imaging is a conservative, portable, non-invasive tool that, being able to visualize the fascial layers [21], has become important in TLF examination [22]. The increase in TLF thickness is a debated parameter to be evaluated during the US examination of TLF. Few researchers have addressed the problem of US TLF thickness evaluation. Langevin et al. [14] showed an increase in perimuscular connective tissue thickness among LBP patients, while Almazán-Polo et al. [23] reported no differences between athletes with and without chronic lumbopelvic pain. For this reason, the purpose of this cross-sectional study was to investigate the difference in TLF thickness at the L3 level of the spine, at a point where the best visibility of the structure is possible, among chronic non-specific LBP patients and a healthy control group. Therefore, we aimed to find out an ultrasonographic parameter or difference that can quantify the TLF’s involvement in chronic non-specific LBP patients.

## 2. Materials and Methods

### 2.1. Study Design

A cross-sectional study based on the Strengthening the Reporting of Observational Studies in Epidemiology (STROBE) statement was conducted [24] in order to compare the US thicknesses of thoracolumbar fascia in the L3 level of the back among lower back pain patients and healthy volunteers. The Helsinki Declaration and human experimentation rules [25] were considered and the Ethics Committee of the University of Padua approved the research. All of the participants were informed prior to inclusion in the project by being provided with a written consent form.

### 2.2. Participants

A total sample of 92 subjects was recruited and divided into two groups: “group 1” comprised 46 subjects with chronic non-specific LBP and “group 2” comprised 46 healthy subjects, from March 2020 to July 2022. Based on the following criteria, the inclusion criteria for group 1 participation consisted of some parameters: patients with non-specific LBP (an evolution > 3 months of LBP) with a history of at least one episode per year of recurrent pain in the past two years and a minimum score of 3 on the visual analogue scale (VAS) [26,27]. Moreover, the latter was measured before US imaging. The inclusion criteria for group 2 were no history of LBP and any limiting pain in daily activities. The exclusion criteria for both groups included a history of spine and lower extremities surgery, spinal deformities, severe pain in the lower back, a history of fracture of the spine and lower extremities, fibromyalgia, balance disorders, and systemic disease such as rheumatological conditions, diabetes, etc. The healthy volunteers in group 2 were matched with the non-specific LBP subjects in terms of age, sex, and BMI. The participants underwent a US examination to assess their US TLF thickness. The enrollment of the subjects was performed by a specialized medical doctor with more than 5 years of experience in physical and rehabilitation medicine.

### 2.3. Ultrasound Examination Measurements

Using a high-resolution device (Edge II, Sonosite, FUJIFILM, Inc. 21919, Bothell, WA, USA) with a frequency range of 6–15 MHz and a screen resolution of 1680 × 1050 pixels, US images were taken at the L3 level of the lumbar spine with a specific US scan protocol. A physician specialist in physical and rehabilitation medicine with 7 years of experience in skeletal muscle US examination and US examination of fasciae carried out the US assessments. A standardized protocol was created and used to assess the TLF for bilateral evaluation. The US system speed of ultrasound was c = 1540 m/s, conventionally used in diagnostic US systems. The US was set to B-mode and depicted a depth of 30 mm. For adequate scans and to reduce surface pressure on the skin, the ultrasonographer used suitable amounts of gel. The probe was placed on the skin as lightly as possible to avoid tissue compression, but it was quite stable to maintain adequate contact between the probe and the skin for consistent images. To eliminate the influence of possible thickness variations, three equidistant points per image for TLF were measured and the resulting values were averaged for analysis. The ultrasonographer followed the same protocol to ensure that each point of the TLF was quantified in the same way. The US beam was kept perpendicular to the TLF because anisotropy artifacts typically affect them. The power and overall gain of the US machine were adjusted to optimize visualization of the fascial planes and t obtain the best scans possible. The US images were frozen and captured.

**Figure 1:**
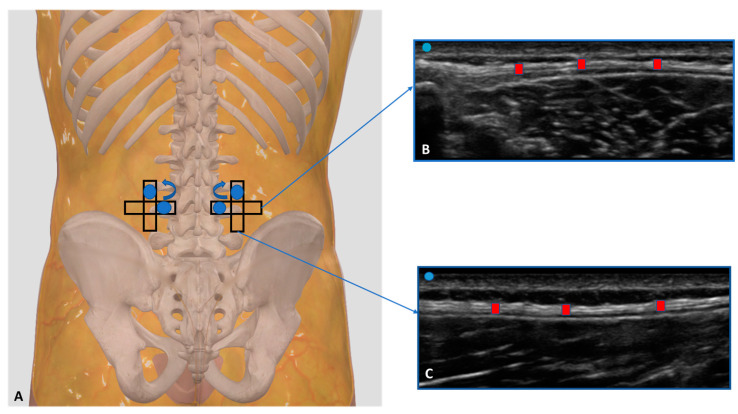
(**A**) Ultrasound measurements protocol of TLF thickness at the L3 level of the lumbar spine. Starting from the transversal axis to the long axis and rotating the probe 90° degree. Probe: black rectangle. (**B**) Transversal/short axis; (**C**) longitudinal/long axis. Red rectangle: TLF thickness.

The ultrasonographer used the short axis beforehand, because it is the best axis to visualize and follow the landmarks correlated with the fascial layers’ visualization imaging used by Pirri et al. [25]; then, the probe was rotated 180° degrees to view at the same point in the longitudinal scan. A specific protocol was defined:

**The L3 level of the lumbar spine:** The patient was relaxed in the prone position and the US transducer was placed parallel to the spine, approximately 2–3 cm lateral to the L3 spinous process (Figure 1). The scans were taken on the short and long axis, rotating the transducer 180° degrees, paying close attention to maintaining the same structure in the center of the US monitoring image, and keeping the probe perpendicular.

All images for each axis were frozen and captured at the end of each assessment, and fascial thickness was measured by Image J analysis software (available online: https://imagej.nih.gov/ij/, access on 11 March 2023). Each image was divided into three regions; in each of them, three points representing the best visibility were measured and averaged. To eliminate the influence of possible thickness variations, three equidistant points for the image were measured, and the resulting values were averaged for analysis. The scanner settings were kept constant during the study.

### 2.4. Statistical Analysis

Statistical analysis was performed using GraphPad PRISM 8.4.2 (GraphPad Software Inc., San Diego, CA, USA), and *p* < 0.05 was always considered as the limit for statistical significance. The resulting effect size was calculated by G Power 3.1 (Universität Düsseldorf: Psychologie) and interpreted according to Cohen’s kappa as small (d = 0.20), medium (d = 0.50), and large (d = 0.80) [28]. For TLF thickness, the effect size was d = 0.57 in our pilot study, as confirmed by another study [29], α error prob = 0.05, power: 1-β err prob = 0.85, and the total sample size was = 90 [28]. Nevertheless, we could include a sample of 92 individuals in our group.

The normality assessment was carried out using the Kolmogorov–Smirnov test. Descriptive statistics were calculated for both groups separately, including measures of central tendency and their dispersion ranges using the mean and standard deviation (SD) to describe parametric data. Finally, a comparative analysis between the chronic non-specific LBP patients group and the healthy volunteers group was performed using an unpaired Student’s *t*-test. The differences in the US-estimated thickness across the different types of axes were statistically analyzed by a paired Student’s *t*-test. In addition, the Pearson’s test was employed for both groups to evaluate the correlation between BMI, weight, height, age, and TLF thickness.

Moreover, a two-way mixed model intra-class correlation coefficient (ICC 3, k) type C was used to assess the intra-rater reliability. The ICC values were interpreted as poor when below 0.5, moderate when between 0.5 and 0.75, good when between 0.75 and 0.90, and excellent when above 0.90 [29].

## 3. Results

A total of 92 subjects (47 females and 45 males) participated in this study. The descriptive data of the sample are summarized in Table 1. No differences were present for BMI, height, weight, and age, showing homogeneity among the groups. Group 1 reported a visual analogue scale (VAS) of 6.8 ± 0.74.

### 3.1. Ultrasound Measurements of the Thoracolumbar Fascia

#### 3.1.1. Group 1 (Chronic Non-Specific LBP Patients)

Regarding Table 2, at the L3 level of the spine, the TLF in the chronic non-specific LBP patients had a mean US thickness of 2.11 ± 0.65 mm (Table 2).

**Figure 2:**
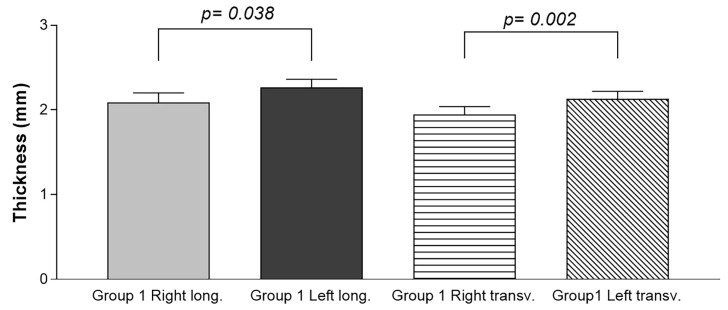
Ultrasound thickness measurements of the TLF in the chronic non-specific LBP patients (Group 1). Long.: longitudinal axis; transv.: transverse axis.

Moreover, the left longitudinal axis thickness was statistically significantly greater than the right longitudinal axis thickness (*p* = 0.038); the same was the case for the left transversal axis vs. the right transversal axis (*p* = 0.002) (Figure 2).

#### 3.1.2. Group 2 (Healthy Volunteers)

In the healthy volunteers, the TFL thickness at the L3 level of the spine was 1.75 ± 0.85 mm (Table 3).

**Figure 3:**
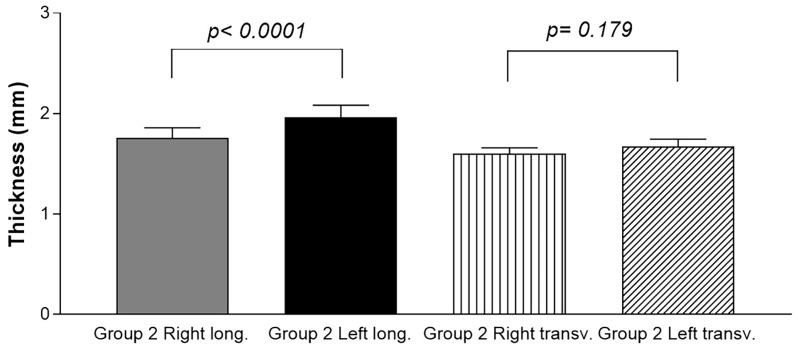
Ultrasound thickness measurements of the TLF in the healthy volunteers (Group 2). Long.: longitudinal axis; transv.: transverse axis.

Moreover, the left longitudinal axis thickness was statistically significantly greater than right longitudinal axis thickness (*p* < 0.0001), while no statistically significant difference was present in the left transverse axis vs. the right transverse axis (*p* = 0.179) (Figure 3).

### 3.2. Ultrasound Measurements of the Thoracolumbar Fascia: Comparison between Group 1 and Group 2

The comparison between the different scans (long. and transv.) between group 1 and group 2 showed a statistically significant difference in the US TLF thickness (Table 4). These differences were present not only for both the longitudinal axis and the transverse axis, but also for both the right and left sides.

### 3.3. Ultrasound Measurements of the Thoracolumbar Fascia Thickness: Comparison between Group 1 and Group 2 for Both the Longitudinal Axis and the Transverse Axis

According to the paired Student’s *t*-test (Table 5), the comparison between the different axes in the US examination of the TLF thickness within each group showed a statistically significant difference between the longitudinal and the transverse axes in group 2 for both sides (Group 2 Left long. vs. Group 2 Left transv.: *p* = 0.001; Group 2 Right long. vs. Group 2 Right transv.: *p* = 0.02) (Table 4). Moreover, in group 2, the TLF had a greater thickness in the longitudinal axis than in the transverse axis (Table 3).

Regarding group, 1 no statistically significant difference was shown (Table 4, Figure 2).

### 3.4. Correlation Ultrasound Measurements and Descriptive Data

According to the correlation analysis, there was a statistically significant correlation between VAS and US TLF thickness in the right transverse axis for group 1 (r = 0.2920; *p* = 0.049).

### 3.5. Intra-Rater Reliability

In addition, the intra-rater reliability was reported as good and excellent. The results for the longitudinal TLF were: left longitudinal (group 1: ICC_3,k_: 0.91; 0.88–0.94—group 2: ICC_3,k_: 0.92; 0.88–0.96) and right longitudinal (group 1: ICC_3,k_: 0.92; 0.88–0.96—group 2: ICC_3,k_: 0.92; 0.88–0.96). In the transverse TLF: left (group 1: ICC_3,k_: 0.88; 0.85–0.90—group 2: ICC_3,k_: 0.88; 0.85–0.90) and right (group 1: ICC_3,k_: 0.88; 0.85–0.90—group 2: ICC_3,k_: 0.88; 0.85–0.90) (Table 6).

## 4. Discussion

Based on our current knowledge, this study may be stated as the first study detailing the TLF thickness at the L3 level of the lumbar spine in chronic non-specific LBP patients compared with healthy volunteers. As has been reported by other studies examining the TLF, the TLF was easily visualized in the longitudinal and transverse axes, appearing with multilayer, linear, and hyperechogenic layers below the subcutaneous tissue [30,31].

**Figure 4:**
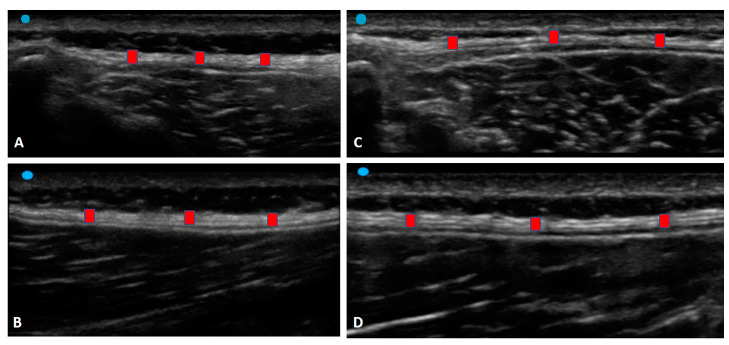
Ultrasound images of the TLF thickness: (**A**) Group 1: transverse axis; (**B**) Group 1: longitudinal axis; (**C**) Group 2: transverse axis; (**D**) Group 2: longitudinal axis. Red square: TLF.

The study’s primary aim was to investigate the difference in the TLF thickness at the L3 level of the lumbar spine in chronic non-specific LBP patients compared with healthy volunteers. An analysis of our results on the TLF thickness showed that in group 1, at the longitudinal and transverse axes, it was thicker (long. = 2.20 ± 0.8 mm; transv.: 2.10 ± 0.61 mm) (Table 2) than group 2 (long. = 1.90 ± 0.8 mm; transv.: 1.65 ± 0.45 mm) (Table 3), showing statistical differences (Table 4, Figure 4).

In light of these findings, the TLF tends to be thicker in the chronic non-specific LBP patients. It remodeled over time in response to repetitive stresses created by pre-existing altered movement patterns due to repetitive motion, habitual posture, and sports [13]. Moreover, the TLF playing an important role in myofascial force transmission [14] can easily change in terms of stiffness and movement impairment, remodelling itself in debilitated tissue that is densified and fibrotic. These results have confirmed, as has been demonstrated by other studies [13,14,15], that in chronic LBP, microinjury and/or inflammation influence nociceptor activation and body movement patterns through a series of interrelated mechanisms that also include aberrant afferent input and maladaptive tissue remodelling [32].

In addition, statistically significant differences in the TLF thickness were evident between the longitudinal and transverse axes within group 2 for both sides (Group 2 Left long. vs. Group 2 Left transv.: *p* = 0.001; Group 2 Right long. vs. Group 2 Right transv.: *p* = 0.02) (Table 4). Pirri et al. [27] showed that expertise in US imaging and identifying anatomical landmarks from a fascial point of view is fundamental, as is the position of the probe and the type of axis; for this reason, we decided to create this protocol for the TLF. Indeed, the latter has a specific basal tension in physiological conditions which lets it perceive the contraction of the underlying muscles due to local mechanoreceptors. This effect permits us to perceive the lateral transmission of force. An alteration to the viscoelastic features of the TLF, which is anatomically in contiguity with it, can modify the field lines of traction within the fascial tissue, creating an alteration in its basal tension. Possibly, we hypothesize that these differences between the longitudinal and transverse axes are evident in healthy volunteers due to normal fascial adaptation to the lateral transmission of force, while in chronic non-specific LBP patients, fascial remodelling creates a situation of blockage of the normal movement with a decrease in fascial adaptation, maintaining the same thickness in the two axes.

Moreover, these findings extend those about the anisotropic behavior of fasciae [33], confirming how a healthy TLF has a good adaptive capacity that is different in multiple directions of movement [34]. According to our data, the patients with chronic non-specific LBP lost TLF anisotropy, with it becoming homogeneously thicker. The aponeurotic fasciae, as with the TLF, are usually more rigid in the longitudinal direction, working as a tendon, connecting different body segments and different muscles, and being more adaptable in the transversal direction [34]. This behavior allows the adaptability of the fasciae to the volume variation of the underlying muscles. In our patients, the US evaluation suggests that the TLF lost this anisotropic behavior, with it becoming thicker and rigid in both directions, a sort of “frozen back”.

**Figure 5:**
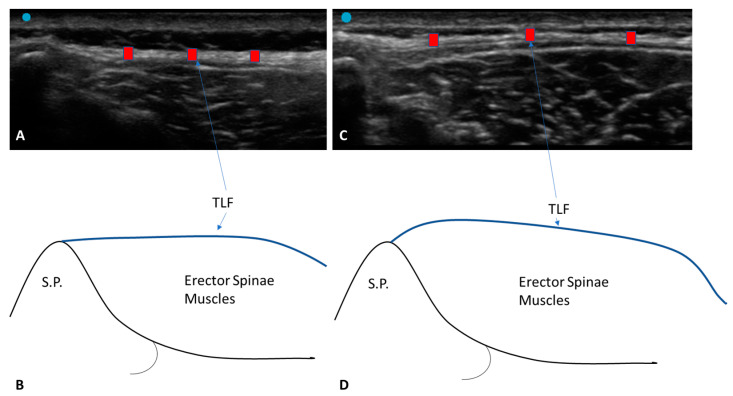
Ultrasound images of the TLF thickness: (**A**) Group 1: transverse axis; (**B**) Group 1: non-tensional force on the TLF; (**C**) Group 2: TLF profile in the transverse axis; (**D**) Group 2: TLF profile in the transverse axis. Red square: TLF.

Myofascial pathways of force have been observed at various levels including between adjacent fibers [35]. The use of advanced magnetization transfer contrast imaging (MRI) has demonstrated the effect of aging but also the effect of disuse on the remodelling of the extracellular matrix by force transmission in the human musculoskeletal system [36]. Indeed, in chronic non-specific LBP patients, some authors have suggested micro-injuries of paraspinal connective tissue and the TLF as possible causes of LBP [37]. Adhesions between TLF layers and epimysium of the erector spinae and multifidus muscles and densification of the TLF are considered determinants of LBP [38]. All of this alters and limits daily movement determining the flaccidity of lumbar segments and altered pattern motion [12]. That being said, the alteration in the muscle activation patterns in chronic non-specific LBP patients modifies the proprioception of these subjects in a cascade (Figure 5).

Furthermore, the correlation between VAS and US TLF thickness in the right transverse axis for group 1 (r = 0.2920; *p* = 0.049) could be explained by the fact that after the TLF becomes densified and sensitized, those free endings will be more and more stimulated when the TLF is strained by the contraction of the muscle. For this reason, a vicious circle of a “frozen back” leads to fascial remodelling that blocks the stretch of free nerve endings to reduce pain, maintaining homogeneous and greater thickness.

The results have confirmed, as has been demonstrated by other studies, that there is good and excellent intra-rater reliability in the US assessment of the deep fasciae, in the case of sonographers with optimal US technical skills and fascial anatomy knowledge [27].

This is the first work to our knowledge to examine and compare the TLF thickness in different axes using US imaging between chronic non-specific LBP patients and healthy volunteers. Future longitudinal studies including a larger number of patients will be able to contribute to our knowledge of the pathophysiology of different thickness patterns. US may also be able to uncover changes that are invisible during clinical examination. Finally, being able to define TLF thickness in fascial dysfunction would facilitate a more targeted approach to treatment.

### Limitation of Study

The low power of the study means that it is not possible to statistically analyze the prevalence of the US findings as well as explain their possible causes, prognostic significance, and therapeutic implications. Moreover, the US examination of TLF morphology greatly depends on the skill of the sonographer as well as the proper setting of the US device.

## 5. Conclusions

US permits an optimal visual examination of the fascial layers in patients with chronic non-specific LBP, with it being a safe, inexpensive, non-invasive, portable, and, most of all, effective instrument that can help clinicians to better understand fascial dysfunction and pathology. In addition, it may reveal changes not highlighted by normal clinical examination. A few of these changes require further investigation because they have not yet been described. To conclude, the study results confirm that in chronic non-specific LBP patients, the TLF is thicker than in healthy volunteers. Moreover, in these patients, no statistically significant difference was found between the longitudinal and transverse axes, which was instead evident in the healthy subjects. The TLF thickness behavior in these patients points out altered fascial remodelling that maintains a homogenous and greater thickness compared to the healthy subjects, a sort of “frozen back”.

## Figures and Tables

**Table 1:** Descriptive data of the samples.

Data	Group 1	Group 2	*p*-Value Group 1 vs. Group 2
Age, y Weight, kg Height, cm BMI, Kg/cm^2^	28.96 ± 10.54 66.22 ± 6.1 168.3 ± 4.83 23.37 ± 5.22	27.09 ± 12.38 70.60 ± 12.20 171.30 ± 6.76 24.03 ± 6.1	*p* = 0.14 *p* = 0.45 *p* = 0.55 *p* = 0.6

Mean ± standard deviation (SD) was applied.

**Table 2:** Ultrasound thickness measurements of the thoracolumbar fascia at the L3 level of the lumbar spine in chronic non-specific LBP patients. Abbreviations: long. = longitudinal scan; trans. = transversal scan.

Descriptive Statistics	Right (long.)	Right (trans.)	Left (long.)	Left (trans.)
Number of values	46	46	46	46
Minimum	0.9	1.2	1.1	1.33
Maximum	5.32	4	3.9	3.8
Mean	2.088	1.948	2.268	2.129
Std. deviation	0.7704	0.6273	0.631	0.6188
Std. error of the mean	0.1136	0.09249	0.09304	0.09124
Coefficient of variation	36.89%	32.2%	27.82%	29.06%

**Table 3:** Ultrasound thickness measurements of the thoracolumbar fascia at the L3 level of the lumbar spine in the healthy volunteers.

Descriptive Statistics	Right (long.)	Right (transv.)	Left (long.)	Left (transv.)
Number of values	46	46	46	46
Minimum	1.01	1.1	1.1	1.1
Maximum	3.9	2.52	4.6	2.84
Mean	1.75	1.6	1.96	1.7
Std. deviation	0.71	0.4	0.81	0.51
Std. error of the mean	0.11	0.1	0.12	0.1
Coefficient of variation	40.43%	24.27%	41.28%	30.28%

Abbreviations: long. = longitudinal scan; trans. = transverse scan.

**Table 4:** Ultrasound measurements comparison between group 1 and group 2. Only statistically significant differences are reported.

Type of Comparison	Mean Diff.	t	*p*-Value
Group 1 Right (long.) vs. Group 2 Right (long.) Group 1 Right (transv.) vs. Group 2 Right (transv.) Group 1 Left (long.) vs. Group 2 Left (long.) Group 1 Left (transv.) vs. Group 2 Left (transv.)	0.3333 0.3450 0.3059 0.4587	2.019 3.085 2.117 3.887	*p* < 0.05 *p* = 0.03 *p* = 0.03 *p* = 0.0003

**Table 5:** Ultrasound measurements comparison between th edifferent axes in the US examination of the TLF thickness within each group. Bold: statistically significant differences.

Type of Comparison	Mean Diff.	t	*p*-Value
Group 1 Left (long.) vs. Group 1 Left (transv.) Group 1 Right (long.) vs. Group 1 Right (transv.) Group 2 Left (long.) vs. Group 2 Left (transv.) Group 2 Right (long.) vs. Group 2 Right (transv.)	−0.1391 −0.1497 0.2920 0.1524	1.922 1.941 3.306 2.347	*p* = 0.06 *p* = 0.08 ***p* = 0.001** ***p* = 0.02**

**Table 6:** Intra-rater reliability of the ultrasound TLF measurements within the different axes of group 1 and group 2.

Type of Axis	ICC
Group 1 Left (long.) Group 1 Left (transv.) Group 1 Right (long.) Group 1 Right (transv.) Group 2 Left (long.) Group 2 Left (transv.) Group 2 Right (long.) Group 2 Right (transv.)	0.91 (0.88–0.94) 0.88 (0.85–0.90) 0.92 (0.88–0.96) 0.88 (0.85–0.90) 0.92 (0.88–0.96) 0.88 (0.85–0.90) 0.92 (0.88–0.96) 0.88 (0.85–0.90)

## Data Availability

The data presented in this study are available upon request from the corresponding author. The data are not publicly available due to privacy.
